# Home visits by neighborhood Mentor Mothers provide timely recovery from childhood malnutrition in South Africa: results from a randomized controlled trial

**DOI:** 10.1186/1475-2891-9-56

**Published:** 2010-11-22

**Authors:** Ingrid M le Roux, Karl le Roux, W Scott Comulada, Erin M Greco, Katherine A Desmond, Nokwanele Mbewu, Mary Jane Rotheram-Borus

**Affiliations:** 1 Philani Child Health and Nutrition Project, Khayelitsha, PO Box 40188, Elonwabeni, Cape Town, 7791, South Africa; 2 Zithulele Hospital, Eastern Cape Zithulele Village, Mqanduli District, 5080, South Africa; 3 Semel Institute for Neuroscience and Human Behavior, University of California Los Angeles 360 Westwood Blvd., Los Angeles, California 90095, USA

## Abstract

**Background:**

Child and infant malnourishment is a significant and growing problem in the developing world. Malnourished children are at high risk for negative health outcomes over their lifespans. Philani, a paraprofessional home visiting program, was developed to improve childhood nourishment. The objective of this study is to evaluate whether the Philani program can rehabilitate malnourished children in a timely manner.

**Methods:**

Mentor Mothers were trained to conduct home visits. Mentor Mothers went from house to house in assigned neighborhoods, weighed children age 5 and younger, and recruited mother-child dyads where there was an underweight child. Participating dyads were assigned in a 2:1 random sequence to the Philani intervention condition (n = 536) or a control condition (n = 252). Mentor Mothers visited dyads in the intervention condition for one year, supporting mothers' problem-solving around nutrition. All children were weighed by Mentor Mothers at baseline and three, six, nine and twelve month follow-ups.

**Results:**

By three months, children in the intervention condition were five times more likely to rehabilitate (reach a healthy weight for their ages) than children in the control condition. Throughout the course of the study, 43% (n = 233 of 536) of children in the intervention condition were rehabilitated while 31% (n = 78 of 252) of children in the control condition were rehabilitated.

**Conclusions:**

Paraprofessional Mentor Mothers are an effective strategy for delivering home visiting programs by providing the knowledge and support necessary to change the behavior of families at risk.

## Introduction

Childhood malnutrition is a serious global problem, causing the deaths of 3.5 million children under 5 years old each year, as well as over a third of the disease burden in this age group [1]. Over one fifth of all children worldwide are underweight [1]. Globally, childhood malnutrition declined somewhat during the 1990's; however, the prevalence of undernourished children in Africa actually increased during that time [2]. In South Africa, 7% of children under 5 die each year; and 12% of under-5 children are underweight; 5% of South African children less than 5 years old suffer from wasting (low weight for height), and over a quarter of under-5 children suffer from stunting (low height for age) [3].

The cascading effects of childhood malnutrition include diminished immune functioning; which leads to greater susceptibility to infection, especially gastrointestinal and respiratory infections; which leads in turn to increased child mortality. Even mild to moderate malnutrition significantly undermines a child's health and chances of survival. A moderately underweight child has a five times higher risk of dying of diarrhea and a four times higher risk of dying of respiratory infections and malaria compared to a child with normal weight [2]. Other consequences are decreased growth and development, including cognitive development [1,2,4]. Children who were malnourished at younger ages demonstrate lower IQ, poor school achievement, and exhibit behavior problems when they reach school age [4]. Long-term malnutrition results in shorter adult height, reduced economic productivity, lifelong impairments in neurocognitive and socioemotional development[5-7] and reduction in the long-term quality of adjusted life years [8].

Recovery from malnutrition can occur with improvements in dietary practices such as conforming more closely to infant feeding guidelines, exclusive breastfeeding in the first 6 months of life, introduction of appropriate solid foods at 6 months, frequent feedings and continuation of breastfeeding for up to 2 years. Other recovery steps include the addition of micronutrients such as Vitamin A, iron, zinc, and iodine, continuation with more nutritious foods after infancy, and preventing diarrhea and infections by avoiding contaminated food, and unhygienic, cold and wet surroundings. South African feeding practices differ significantly from what is recommended. For example, only 8% of babies younger than 6 months are exclusively breastfed, and fewer than half of babies 6-9 months old have a diet combining breastfeeding with complementary foods [9]. Critical components of the recovery pathway include taking advantage of available resources and improving mothers' feeding and caring practices.

The Philani child health and nutrition program was developed in the townships surrounding Cape Town, South Africa, to provide this assistance to mothers of underweight and at-risk children. Philani incorporates elements used successfully in other child health and nutrition programs, including peer role models, peer educators and home visits during the important early years of a child's life. Role models, or "positive deviants" (mothers whose children are thriving despite living in the same resource-poor settings as their peers) are a key component of the Hearth model for malnutrition recovery that has been used in Haiti, Vietnam, and Bangladesh [10-13]. Peer nutrition educators have been used in a variety of settings such as the U.S. Expanded Food and Nutrition Education Program, which improves nutrition knowledge and practices among a half-million low-income participants each year [14-16]. Nurse and paraprofessional home visiting has also been used in the U.S. to provide social support, health education, and practical assistance to families with very young children [17-22]

The Philani program uses positive deviant "mentor mothers" to provide nutrition education and support to parents in an outreach program of home visitation. In addition to nutrition, the program also addresses related issues such as securing government assistance where appropriate (making it easier to afford more and better food), increasing mother-child bonds, improving hygiene and protection against cold and wetness, improving feeding practices, and reducing abuse and neglect. An immediate goal of the Philani program is to rehabilitate malnourished children to weights that are appropriate for their ages, as quickly as possible, to counteract the deleterious effects of remaining undernourished. The longer a child remains malnourished, the longer he or she remains at risk of infection, developmental deficits, and other adverse consequences. An earlier program evaluation demonstrated that malnourished children in the Philani program recovered more weight during a one-year period compared to children in the control group (le Roux IM, le Roux K, Comulada WS, Desmond KA, Rotheram-Borus MJ.: Home visits by neighborhood mentor mothers to improve children's nutrition in South Africa, in submission). The purpose of this paper is to evaluate how quickly the Philani program could bring a second cohort of malnourished children up to healthier weights, when compared to community controls.

## Methods

During the period of 2006-2007, 65 neighborhoods of about 800 households each were identified in three Xhosa townships surrounding Cape Town, South Africa. The neighborhoods contained both formal settlements (government housing with an address and onsite water and sewage), site-and-service plots (plots of land where residents can build a home, with some access to water and sanitation facilities), and informal settlements (shacks or temporary structures that rarely have water or access to sanitation on the premises and are not on a specified plot of land). A mentor mother (MM) was recruited for each neighborhood using a variety of sources such as referrals by local community leaders, or by open application. Nominees were interviewed by supervisors for the Philani program. MMs chosen for the program had children who were thriving; they demonstrated strong communication and interpersonal skills, commitment to community service and showed an organized and disciplined approach to tasks. Thriving children and organizational skills were confirmed by home observations of the MM's household, where Philani supervisors ascertained whether the home was organized, children were monitored, and healthy food was available. MMs who were selected received training in child health, nutrition and other related topics. After training, one MM was assigned to each neighborhood, typically based on residential proximity. MMs received a stipend of $US 130/month from Philani to deliver home-based interventions and were expected to work four hours a day.

**Figure 1:**
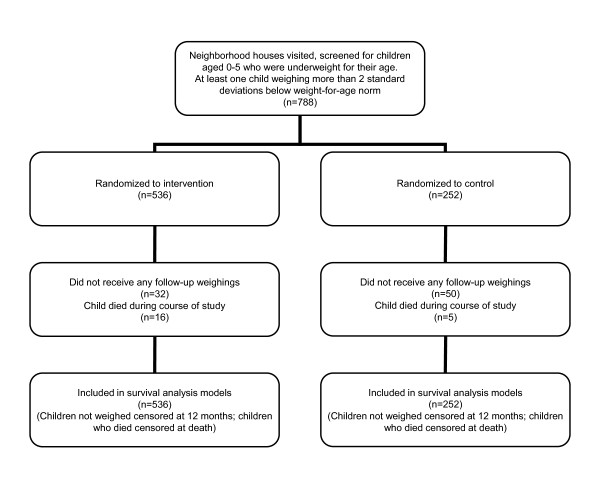
Flow of participants through the study.

The flow of participants through the study is shown in Figure 1. MMs visited every home in their neighborhoods, identifying and weighing each child aged 5 or younger. The child's age and weight were plotted on a growth chart containing age-appropriate norms. Any child weighing less than 2 standard deviations below his or her weight-for-age norm (<-2SD) was classified as malnourished; this included all newborns weighing less than 2500 grams at birth. Any household with at least one malnourished or low birth weight child was invited to participate in the study. If there were multiple malnourished children in a household, all were followed but only one was randomly selected to be included in the analysis. Over 12 months, the 65 MMs recruited 788 mothers and their babies or children aged 5 or under. The mother-child unit is referred to as a *dyad*. Assignment to the intervention condition was based on a sequence decided *a priori *for every three dyads. Because the likelihood of benefits from the intervention far exceeded any chance of harm, the allocation ratio of intervention to control was 2:1 [23,24]. Two out of three dyads (in random order) were assigned to the Philani intervention condition (n = 536). The third dyad became a control case (n = 252). The MMs were given randomly sequenced numbered folders marked I for intervention and C for controls and supervisors made sure the folders were allocated in the correct order. After data collection was completed, dyads assigned to the control condition were given the option of joining the Philani nutrition intervention program.

This study was approved by the UCLA Office of the Human Research Protection Program (#G07-02-033) and the Stellenbosch University Health Research Ethics Committee (#N08/08/218).

### Intervention Description

Mentor Mothers received four phases of training: 1) watching experienced MMs implement the intervention in an inspiring manner, learning how to approach a family and build trust; 2) attending a month of training that covered nutrition; basic child health including HIV and TB, weighing of babies and completion of growth charts; how to recognize signs of abuse and crisis situations; and how to encourage depressed mothers to be more active and engaged with their children; 3) learning how to help mothers bond with their children and improve the consistency of healthy daily routines; and 4) implementing their first round of home visits independently in their neighborhoods.

An essential part of the intervention is for the MM to create a respectful and caring relationship with the mother/parent. Throughout the Philani experience, we have found that changing behavior is not possible without such trusting relationships. The MM who is a positive deviant has developed coping mechanisms which have made it possible for her to raise healthy children and a key component of the intervention is for her to share those coping mechanisms with other mothers. These might include initiating and maintaining breastfeeding; introducing solids correctly; feeding frequently but also creating good sleeping habits; providing organization, discipline and structure in the home; protecting the child from sources of infection, accidents and trauma; and seeking care when needed. A successful intervention will see the child gaining weight rapidly until fully rehabilitated, with a significant decrease in episodes of infection. With a healthy growing child the incidence of maternal depression will decrease and the bonding between mother and child will improve.

A supervisor accompanied each MM at least one day a month on a random schedule to ensure that implementation proceeded as planned. The supervisor collaborated with the MM in problem-solving and generating action plans when problems occurred in the field. Each MM and supervisor built a list of clinics and hospitals for referrals and a strategy for providing services relating to mental health issues (particularly post-partum depression), partner abuse, and legal problems. The quality of the implementation was monitored by reviewing the forms completed at each home visit, monitoring visitation patterns, collecting observations by supervisors, and brief ratings of home visits by the supervisors.

A typical MM visit at each home lasted 20 to 60 minutes. During the visits, the MM weighed the participating child and discussed his progress with the mother. The MM also made sure the mother had the social grants she might be entitled to, and that the mother understood proper nutrition and hygiene. MMs stressed the importance of breastfeeding, the proper time to introduce solids, frequent feeding, and a mixed diet including vegetables and fruit. She checked to see if immunizations were up to date and that the child was dewormed. In each MM's caseload there was likely to be one emergency a week (e.g., a child would be ill with high fever, have difficulty breathing, or appear severely dehydrated). These cases were brought to the Philani Health Clinic or the local public health clinic to receive immediate attention. MMs did not distribute food supplements.

### Measures

The following data was collected by MMs at the time of recruitment, from dyads in both the intervention and control arms of the study:

○ Children's background characteristics. Mothers reported on several characteristics of the child enrolled in the study: age, gender, birth weight, and whether or not the child was already enrolled in a nutrition program.

○ Mothers' background characteristics. Mothers also reported whether any of their children had died, whether they were employed, whether they were receiving any government grants, where they were born, and number of years living in the Cape Town area.

○ Housing/living situation. Mothers reported housing conditions (classified as formal, site and service, or informal), number of adults living in the household, whether there was water onsite at the home, whether there was a flush toilet, and whether they had had to reduce or skip meals due to lack of money. MMs reported two subjective measures for the mothers' living conditions: overall smell (pleasant, neutral, or poor) and hygiene (good, average, or poor).

Children in the control condition were weighed by MMs at baseline, and at 3, 6, 9, and 12-month follow-ups. Children in the intervention condition were also weighed by MMs at baseline, and at each intervention home visit. Rehabilitation to an acceptable weight was indicated by achieving a weight-for-age Z-score (WAZ) [25] that was above the cutoff for study eligibility (>-2SD), i.e., above the third percentile of weight-for-age norms. Time to rehabilitation was noted at the first assessment at which the child reached the target weight.

### Statistical Methods

We compared demographic and household characteristics of the dyads across intervention conditions at recruitment. We also compared dyads followed over time vs. those with no follow-ups. Chi-square tests and *t *tests were conducted for categorical and continuous measures, respectively. Where appropriate, Fisher's exact test was conducted on categorical measures with sparse cell counts and the Wilcoxon two-sample test was conducted on continuous measures with skewed distributions.

We compared the time to rehabilitation between the intervention and control conditions using discrete time survival analysis models [26]. Discrete-time versus continuous-time models were used to capture the discrete nature of the follow-up intervals. That is, when normal weight was achieved, we knew that it had occurred between the current and previous assessment, but we did not know the exact time. Therefore, a child rehabilitated any time between the baseline and 3-month assessments was considered to be rehabilitated at 3 months and a child rehabilitated after 3 months but before the 6-month assessment was considered to be rehabilitated at 6 months. Rehabilitation times were coded similarly at the 9 and 12 month assessments. Using this technique, the probability of rehabilitation at a given assessment is conditioned on the child surviving and not being rehabilitated prior to that period. A logistic framework is used, modeling the log odds of rehabilitation as a function of time period and other covariates in the model.

There were three possible outcomes for children during the study period: 1) rehabilitation (i.e., achieving an acceptable weight); 2) death, at which point they no longer contributed to person-period time (i.e., observations beyond death were censored); or 3) the child did not reach normal weight by the final follow-up and was censored at 12 months. Because of the possibility of intermittency in the follow-up weighings (i.e., missing an earlier weight but not missing later ones), there was no censoring for loss to follow-up. For time periods where there was missing data (i.e., a weight was not recorded), it was assumed that rehabilitation had not occurred. To account for dyads for whom no follow-ups were obtained, models were run two ways. In the first, it was assumed that rehabilitation did not occur, and the child was censored at 12 months. In the second, these dyads were removed from the analysis. Results were nearly identical under both approaches, and the results from the analyses that assumed no rehabilitation are presented here. Discrete-time survival models were implemented in SAS software version 9.1 (SAS Institute Inc., Cary, NC, USA) [26].

Models included covariates for randomized intervention condition assignment, classified as intervention or control; discrete time interval, classified as 3, 6, 9, or 12-months; intervention-by-time interval interactions to test the intervention effect; and background characteristics, including child age, gender, and living conditions.

## Results

Table 1 shows the demographic and background characteristics of mother-child dyads at recruitment. Half of the dyads lived in informal housing (52%) and had access to a flush toilet (55%). MMs reported living conditions of the dyads to have a pleasant or neutral smell (92%), but less than a third of homes were thought to have good hygiene (32%). A little less than a fifth of the children were supported by a nutrition program (19%) and half of the children were of low birth weight (53%). On average, children in the intervention condition were a few months younger (17.3 months at recruitment vs. 21.2; *t *= 3.29, *P *< .01), and weighed a kilogram less (mean = 6.8 vs. 7.8; *t *= 4.22, *P *< .01). WAZ was lower among intervention children (-3.4 vs. -3.1, t = 2.05, *P *< .05). Among those dyads where birth weight was known, a greater percentage of children in the intervention condition had been of low birth weight (56% vs. 46%; *P *< .05). None of the other demographic or background characteristics differed significantly across intervention conditions.

**Table 1:** Baseline characteristics of children and mothers enrolled in Philani intervention and control groups

	Control group (n = 252)		Philani intervention (n = 536)		Total (n = 788)		
	n	%	n	%	n	%	
**Child characterisics**							
Mean age at admission (months), SD	21.2	16.6	17.3	15.3	18.5	15.9	**
Female gender	163	64.7	316	59.1	479	60.9	
Low birth weight (N = 657)	92	46.2	258	56.3	350	53.3	*
Mean weight at admission (kilograms), SD	7.8	3.0	6.8	3.1	7.1	3.1	**
Mean weight-for-age Z-score (WAZ), SD	-3.1	1.9	-3.4	1.0	-3.3	1.4	*
Supported by nutrition program	38	15.1	109	20.4	147	18.7	
							
**Mother characteristics**							
Has deceased children	6	2.4	21	3.9	27	3.4	
Employed	22	8.9	56	10.5	78	10	
Received any grants	176	70.4	341	63.9	517	65.9	
Born outside of Cape Town	215	86.4	453	85.0	668	85.4	
Mean number of years in Cape Town, SD	10.1	7.5	10.0	7.1	10.0	7.2	
							
**Housing/living situation**							
*Reported by mother*							
Housing description							
Formal	76	30.3	160	30.2	236	30.3	
Site & service	47	18.7	92	17.4	139	17.8	
Informal	128	51.0	277	52.4	405	51.9	
Mean number of adults living in household, SD	4.6	2.6	4.6	2.7	4.6	2.7	
Water on site	119	47.4	254	48.1	373	47.9	
Flush toilet vs. bucket/nothing	138	55.9	291	55.1	429	55.4	
Cut size of meals due to lack of money							
Child(ren)'s meals	70	28.0	133	25.1	203	26.0	
Adult(s) meals	91	36.6	170	32.1	261	33.6	
							
*Reported by interviewer*							
No foul odor on property	226	91.5	487	92.1	713	91.9	
Good hygeine	79	31.7	167	31.5	246	31.5	

* *P *< .05, ** *P *< .01

We compared characteristics from Table 1 between dyads we were unable to follow after recruitment (10%; 82 of 788) vs. dyads with at least one follow-up (90%; 706/788). Dyads without follow-up were more likely to have older children (25.2 vs. 17.8 months, *t *= 4.08, *P *< .01), less likely to have the child supported by a nutrition program (10% vs. 20%, χ^2 ^= 4.80, *P *= .03), more likely to have a mother who was employed (18% vs. 9%, χ^2 ^= 6.11, *P *= .01), and less likely to have a flush toilet versus less modernized toilet facilities (44% vs. 57%, χ^2 ^= 4.36, *P *= .04). Dyads in the control condition were more likely to miss follow-ups compared to those in the intervention condition (20% vs. 6%; χ^2 ^= 35.38, *P <*.01). None of the other demographic or background characteristics differed significantly by whether or not dyads had follow-ups.

**Figure 2:**
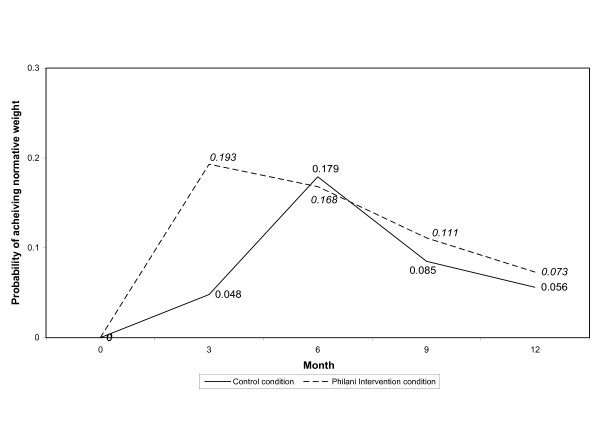
**Adjusted hazard function for achieving normative weight stratified by intervention condition. **The solid line represents children's probability of achieving normal weight over 12 months in the control condition. The dashed line represents children's probability of achieving normal weight in the Philani intervention condition over 12 months.

**Figure 3:**
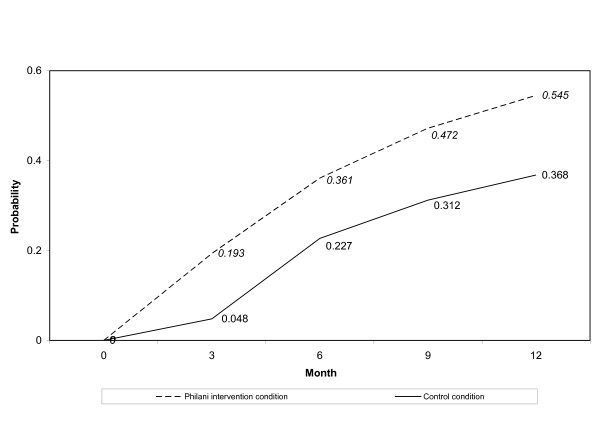
**Cumulative adjusted probability for achieving normative weight stratified by intervention condition. **The solid line represents the control condition probability over 12 months, while the dashed line represents the Philani intervention condition probability over 12 months.

Over 12 months, low-weight babies and children in the intervention condition were rehabilitated in a shorter period of time than those in the control condition. This is shown graphically in two ways. Figure 2 shows the probability of rehabilitation for each time period. The odds of rehabilitation by 3 months were almost 5 times higher in the intervention compared to the control (OR = 4.74, 95% CI = 2.47-9.09). By 6 months, the odds of rehabilitation across intervention conditions were similar (OR = .90, 95% CI = .58-1.41) and remained that way at 9 months (OR = 1.31, 95% CI = .69-2.48) and 12 months (OR = 1.27, 95% CI = .56-2.86). Figure 3 shows the cumulative probability of rehabilitation at each time period by intervention condition. The number of infants/children who remained underweight at the beginning of each time interval and the number rehabilitated during the time interval are shown in Table 2. A higher percentage of children in the intervention condition were rehabilitated over the course of the study (43%; n = 233 of 536) compared to the control condition (31%; n = 78 of 252; χ^2 ^= 11.24, *P *< .01).

**Table 2:** Number of children remaining underweight or being rehabilitated in each time interval

	Number of non-censored* children below normative weight at beginning of time interval			
	(Number of children rehabilitated during time interval)			
	Baseline to 3 months	3 to 6 months	6 to 9 months	9 to 12 months
Control	252 (13)	237 (39)	195 (15)	180 (11)
Philani intervention	536 (102)	426 (70)	351 (40)	308 (21)
Total	788 (115)	663 (109)	546 (55)	488 (32)

* Censoring occurred at a child's death (n = 21; mortality did not differ by intervention condition).

## Discussion

Although children in the intervention condition were more underweight initially, we found that more intervention children were rehabilitated and sooner compared to children in the control condition. The Philani program significantly reduced the amount of time that malnourished children remained underweight compared to malnourished children in the standard care condition. In addition, the percentage of children who had not been rehabilitated within a year was significantly lower in the intervention condition (57%, 303/536) compared to controls (69%, 174/252). This is an important finding, as poor nutrition is one of the major determinants of long-term health and is consistently linked to poor cognitive and developmental outcomes over the lifespan [4-7].

A limitation of this study is that we were unable to obtain consistent measures of infant length or child height. For very young children, it is difficult to obtain accurate measurements even under good conditions [27,28]. In these home visits, often in shacks, it was not possible. As a result, we are unable to determine whether the recovery of malnourished children due to the Philani intervention was in weight alone. Although the intervention should be successful in averting the considerable short term risks of malnutrition, the effect on long-term development is not known. Research on childhood malnutrition has shown that recovery programs involving diets that are heavy in energy and protein may not be the best approach for long-term growth: children in these programs gain weight, but it is often through adding fat, rather than height and lean muscle tissue; rapid weight gain in this manner can lead to health problems later in life [29-32]. Given the high prevalence of stunting among South African children under 5 years old (over 25%), nutrition that promotes healthy linear growth is of paramount importance[33].

Several characteristics of the Philani program give us reason to believe that catch-up attributable to this intervention will not lead to future obesity and related health problems. The children were not given high energy food supplements; instead, mothers were encouraged to breastfeed infants, were taught about healthy nutrition and lifestyle change, and were aided in identifying resources to help pay for more nutritious foods. These dietary changes are likely to promote healthy growth, not just add fat tissue. Children were intervened with at a young age (mean age at admission was 18 months); recovery in length appropriate to age is most likely if a child's nutrition can be improved before he is 2-3 years old [29] and risks of accelerated weight gain for undernourished babies is less likely if the child recovers by age 12-18 months [34-36]. Future research should include measures of height/length, to confirm that the Philani intervention is successful in combating both stunting and wasting in malnourished children. At the same time, it must not be forgotten that achievement of normal weight remains a priority in the townships of South Africa, where adequate nutrition remains scarce and the immediate health consequences of malnutrition take precedence.

A second limitation of the study relates to the procedures for assignment to intervention or control status. Although the randomization sequence in this study was determined *a priori *by the research team, the protocol was administered by MMs. The fact that children in the intervention arm of the study were significantly younger and more underweight indicates that the MMs may not have adhered to the assignments, but rather steered needier children into the immediate intervention. The direction of any bias caused by this deviation from random assignment is likely to be against the detection of an intervention effect: to the extent that children in the intervention had lower WAZ scores, they had further to go to achieve normative weights. Regardless, future research should use procedures that cannot be tampered with, such as neighborhood rather than individual assignment.

A final limitation is that 10% of children were not weighed during the follow-up period. The direction of bias that might be caused by this is not clear, as characteristics of those with and without follow-ups do not indicate clear-cut differences in vulnerability. Results shown are from models that assume that those without follow-ups were not rehabilitated; since more controls than intervention dyads were missing follow-ups, this may imply that estimates of the intervention effect are biased upward. However, models run both including and excluding dyads with no follow-ups produced the same results.

In conjunction with findings from the first Philani study (le Roux IM, le Roux K, Comulada WS, Desmond KA, Rotheram-Borus MJ.: Home visits by neighborhood mentor mothers to improve children's nutrition in South Africa, in submission), our data indicate that paraprofessional MMs can deliver effective home visiting programs, providing the knowledge and support necessary to change health outcomes among families at risk. The objectives of malnutrition recovery programs are to promote catch-up growth, prevent illness and death directly caused by nutritional deficits, improve overall health and the ability to withstand infection, and promote healthy physical and mental development [31]. The timely recovery achieved by very young children in the Philani program is very promising. The supervision and encouragement provided by MMs is of great importance in this challenging work. As has been demonstrated with the Hearth model [9-13], peer nutrition [14-18], and home visiting programs [19-21], community peers can provide a sustainable mechanism for addressing health needs in low resource settings.

## Competing interests

The authors declare that they have no competing interests.

## Authors' contributions

ILR, KLR and MJR supervised the study. The ILR and KLR supervised the intervention and the acquisition of data. WSC, EMG, and KAD designed and conducted the statistical analyses, and interpreted the results. All authors contributed to the writing of the paper. All authors read and approved the final manuscript.

## References

[B1] Black RE Allen LH Bhutta ZA Caulfield LE de Onis M Ezzati M Mathers C Rivera J for the Maternal and Child Undernutrition Study Group Maternal and child undernutrition: global and regional exposures and health consequences Lancet 2008 9 243 260 10.1016/S0140-6736(07)61690-0 18207566

[B2] Blössner M deOnis M Malnutrition: quantifying the health impact at national and local levels Title IV Series 2005 Geneva: WHO Document Production Services [Prüss-Üstün A, Campbell-Lendrum D, Corvalán C, Woodward A (Series Editors): *Environmental Burden of Disease Series*, no. 12.]

[B3] UNICEF - South Africa - Statistics http://www.unicef.org/infobycountry/southafrica_statistics.html

[B4] Grantham-McGregor S A review of studies of the effect of severe malnutrition on mental development Journal of Nutrition 1995 9 Suppl 8 2233S 2238S 7542705 10.1093/jn/125.suppl_8.2233S

[B5] Grantham-McGregor S Cheung YB Cueto S Glewwe P Richter L Strupp B the International Child Development Steering Group Developmental potential in the first five years for children in developing countries Lancet 2007 9 60 70 10.1016/S0140-6736(07)60032-4 17208643 PMC2270351

[B6] Mason JB Bailes A Mason KE Yambi O Jonsson U Hudspeth C Hailey P Kendle A Brunet D Martel P AIDS, drought, and child malnutrition in southern Africa Pub Health Nutr 2005 9 551 563 10.1079/PHN2005726 16236184

[B7] Stanfield JP Some aspects of the long-term effects of malnutrition on the behaviour of children in the Third World Proc Nutr Soc 1993 9 201 210 10.1079/PNS19930052 8493266

[B8] Zere E McIntyre D Inequities in under-five child malnutrition in South Africa Int J Equity Health 2003 9 7 10.1186/1475-9276-2-7 14514357 PMC201028

[B9] South Africa Department of Health, Medical Research Council, OrcMacro South Africa Demographic and Health Survey 2003 Full Report 2007 Pretoria: Department of Health

[B10] United Nations University Press The positive deviance approach to improve health outcomes: experience and evidence from the field Food and Nutrition Bulletin 2002 9 Suppl 4 12503225

[B11] Wollinka O Keeley E Burkhalter BR Bashir N eds Hearth Nutrition Model: Applications in Haiti, Vietnam, and Bangladesh 1997 Published for the U.S. Agency for International Development and World Relief Corporation by the Basic Support for Institutionalizing Child Survival (BASICS) Project. Arlington, VA

[B12] Sternin M Sternin J Marsh D Wollinka O, Keeley E, Burkhalter BR, Bashir N Rapid, sustained childhood malnutrition alleviation through a "positive deviance" approach in rural Vietnam: Preliminary findings Hearth Nutrition Model: Applications in Haiti, Vietnam, and Bangladesh 1997 Published for the U.S. Agency for International Development and World Relief Corporation by the Basic Support for Institutionalizing Child Survival (BASICS) Project. Arlington, VA

[B13] Marsh DR Schroeder DG Dearden KA Sternin J Sternin M The power of positive deviance BMJ 2004 9 1177 1179 10.1136/bmj.329.7475.1177 15539680 PMC527707

[B14] About EFNEP http://www.csrees.usda.gov/nea/food/efnep/about.html

[B15] Arnold CG Sobal J Food practices and nutrition knowledge after graduation from the Expanded Food and Nutrition Education Program (EFNEP) Journal of Nutrition Education 2000 9 3 130 138 10.1016/S0022-3182(00)70540-1

[B16] Burney J Haughton B EFNEP: A nutrition education program that demonstrates cost-benefit Journal of the American Dietetic Association 2002 9 1 39 45 10.1016/S0002-8223(02)90014-3 11794500

[B17] Olds DL Kitzman H Cole R Robinson J Sidora K Luckey DW Henderson CR Jr Hanks C Bondy J Holmberg J Effects of nurse home-visiting on maternal life course and child development: age 6 follow-up results of a randomized trial Pediatrics 2004 9 1550 9 10.1542/peds.2004-0962 15574614

[B18] Olds DL Robinson J O'Brien R Luckey DW Pettitt LM Henderson CR Jr Ng RK Sheff KL Korfmacher J Hiatt S Talmi A Home visiting by paraprofessionals and by nurses: a randomized, controlled trial Pediatrics 2002 9 3 486 96 10.1542/peds.110.3.486 12205249

[B19] Olds DL Robinson J Pettitt L Luckey DW Holmberg J Ng RK Isacks K Sheff K Henderson CR Jr Effects of home visits by paraprofessionals and by nurses: age 4 follow-up results of a randomized trial Pediatrics 2004 9 1560 1568 10.1542/peds.2004-0961 15574615

[B20] Gomby DS Culross PL Behrman RE Home visiting: Recent program evaluations-analysis and recommendations The Future of Children 1999 9 1 4 26 10.2307/1602719 10414008

[B21] Sweet MA Appelbaum MI Is home visiting an effective strategy? A meta-analytic review of home visiting programs for families with young children Child Development 2004 9 5 1435 1456 10.1111/j.1467-8624.2004.00750.x 15369524

[B22] Olds DL Sadler L Kitzman H Programs for parents of infants and toddlers: Recent evidence from randomized trials Journal of Child Psychology and Psychiatry 2007 9 3 355 391 10.1111/j.1469-7610.2006.01702.x 17355402

[B23] Sackett D Haynes RB, Sackett DL, Guyatt GH, Tugwell P The tactics of performing therapeutic trials Clinical epidemiology: how to do clinical practice research 2006 3 Philadelphia, PA: Lippincott Williams & Wilkins

[B24] Friedman LM Furberg CD DeMets DL Fundamentals of Clinical Trials 1981 Boston, MA: Wright PSG

[B25] Kuczmarski RJ Ogden CL Grummer-Strawn LM CDC growth charts: United States. Advance data from vital and health statistics (report no. 314) 2000 Hayattsville, Md: National Center for Health Statistics

[B26] Allison PD Survival Analysis Using the SAS System: A Practical Guide 1995 Cary, NC: SAS Institute Inc

[B27] Johnson TS Engstrom JL Gelher DK Intra- and interexaminer reliability of anthropometric measurements of term infants Journal of Pediatric Gastroenterology and Nutrition 1997 9 5 497 505 10.1097/00005176-199705000-00001 9161941

[B28] Corkins MR Lewis P Cruse W Gupta S Fitzgerald J Accuracy of infant admission lengths Pediatrics 2002 9 6 1108 1111 10.1542/peds.109.6.1108 12042550

[B29] Uauy R Kain J The epidemiological transition: need to incorporate obesity prevention into nutrition programmes Public Health Nutrition 2002 9 1A 223 229 10.1079/PHN2001297 12027288

[B30] Golden MH Proposed recommended nutrient densities for moderately malnourished children Food and Nutrition Bulletin 2009 9 3 S267 S342 19998863 10.1177/15648265090303S302

[B31] Eriksson JG Forsén T Tuomilehto J Winter PD Osmond C Barker DJP Catch-up growth in childhood and death from coronary heart disease: longitudinal study BMJ 1999 9 427 431 9974455 10.1136/bmj.318.7181.427 PMC27731

[B32] Hemachandra AH Howards PP Furth SL Klebanoff MA Birth weight, postnatal growth, and risk for high blood pressure at 7 years of age: results from the Collaborative Perinatal Project Pediatrics 2007 9 6 1264 1270 10.1542/peds.2005-2486 17545358

[B33] Martins VJB Martins PA das Neves J Sawaya AL Children recovered from malnutrition exhibit normal insulin production and sensitivity British Journal of Nutrition 2008 9 297 302 10.1017/S0007114507793959 17651520

[B34] Lucas A Fewtrell MS Cole TJ Fetal origins of adult disease - the hypothesis revisited BMJ 1999 9 245 249 10417093 10.1136/bmj.319.7204.245 PMC1116334

[B35] Eriksson JG Forsén T Tuomilehto J Osmond C Barker DJP Early growth and coronary heart disease in later life: longitudinal study BMJ 2001 9 949 955 10.1136/bmj.322.7292.949 11312225 PMC31033

[B36] Barker DJP Bagby SP Developmental antecedents of cardiovascular disease: a historical perspective Journal of the American Society of Nephrology 2005 9 2537 2544 10.1681/ASN.2005020160 16049070

